# 

**Published:** 1897-02

**Authors:** 


### CONTENTS.

COMMUNICATIONS.

Dental Legislation.... 53
The Defense of Theory.... 62
Some Reasons why we Fail with Cataphoresis.... 66
A Summary of my Experience with Cataphoresis.... 72
Relative Efficiency of Various Current-Controllers for Cataphoresis 76
The Galvanic Current.... 92

SELECTIONS.

Consumption.... 71
Extraction of Teeth and Facial Paralysis.... 100
A Cheap Disinfectant.... 101

OBITUARY.

In Memoriam—William Newton Morrison, D. D. S.... 101

